# Prevalence of distressing symptoms in hospitalised patients on medical wards: A cross-sectional study

**DOI:** 10.1186/1472-684X-7-16

**Published:** 2008-09-23

**Authors:** Katrin Ruth Sigurdardottir, Dagny Faksvåg Haugen

**Affiliations:** 1 Sunniva Clinic for Palliative Medicine, Haraldsplass Deaconal Hospital, N-5008 Bergen, Norway; 2 Regional Centre of Excellence for Palliative Care, Western Norway, Haukeland University Hospital, N-5021 Bergen, Norway; 3 Department of Internal Medicine, Haukeland University Hospital, N-5021 Bergen, Norway; 4 Department of Cancer Research and Molecular Medicine, Faculty of Medicine, Norwegian University of Science and Technology, N-7006 Trondheim, Norway

## Abstract

**Background:**

Many patients with advanced, serious, non-malignant disease belong to the population generally seen on medical wards. However, little research has been carried out on palliative care needs in this group. The aims of this study were to estimate the prevalence of distressing symptoms in patients hospitalised in a Department of Internal Medicine, estimate how many of these patients might be regarded as palliative, and describe their main symptoms.

**Methods:**

Cross-sectional (point prevalence) study. All patients hospitalised in the Departments of Internal Medicine, Pulmonary Medicine, and Cardiology were asked to do a symptom assessment by use of the Edmonton Symptom Assessment System (ESAS). Patients were defined as "palliative" if they had an advanced, serious, chronic disease with limited life expectancy and symptom relief as the main goal of treatment.

**Results:**

222 patients were registered in all. ESAS was completed for 160 patients. 79 (35.6%) were defined as palliative and 43 of them completed ESAS. The patients in the palliative group were older than the rest, and reported more dyspnea (70%) and a greater lack of wellbeing (70%). Other symptoms reported by this group were dry mouth (58%), fatigue (56%), depression (41%), anxiety (37%), pain at rest (30%), and pain on movement (42%).

**Conclusion:**

More than one third of the patients in a Department of Internal Medicine were defined as palliative, and the majority of the patients in this palliative group reported severe symptoms. There is a need for skills in symptom control on medical wards.

## Background

Populations in developed countries are ageing, and an increasing number of persons live with the effects of serious chronic illnesses towards the end of life. Most deaths in European and other developed countries occur in people more than 65 years old [1]. In 2005, there were 41,152 deaths registered in Norway, 83.5% occurred in people aged above 65, and cardiovascular disease (34.4%) and cancer were the causes of more than 60% of all deaths [2]. The top five predicted causes of death for 2020 are heart disease, cerebrovascular disease, chronic respiratory disease, respiratory infections, and lung cancer [3]. Most of the patients with advanced, serious, non-malignant disease belong to the population generally seen on medical wards. None the less, comparatively little research has been carried out on their needs for palliative care. To our knowledge, no survey on the prevalence of distressing symptoms in an internal medicine population has been performed in Norway, and the literature on this topic is generally scarce. The present study was planned to get an impression of the palliative care needs of a general medical inpatient population. In addition, the results would be useful when planning a palliative care service for our regional university hospital.

## Methods

This was a cross-sectional (point prevalence) study performed at Haukeland University Hospital on 17th February 2005. Haukeland University Hospital is a regional teaching hospital with 1100 beds and about 200 admissions daily.

All patients hospitalised in the Departments of Internal Medicine, Pulmonary Medicine, and Cardiology (216 beds in all) were asked to do a symptom assessment by use of a slightly modified ESAS (Edmonton Symptom Assessment System) questionnaire [4], filled in by the patients themselves or by interview. All patients received written and oral information about the study and gave informed consent.

The ESAS questionnaire evaluates nine common symptoms in palliative care patients as well as general wellbeing [4-6]. Numerical rating scales (NRS) from 0 to 10 are used to describe symptom severity, 0 indicating no symptom and 10 indicating worst possible distress. The system has been widely used in palliative care settings [7]. In the present study, scores < 3 on the scales were defined as normal or no symptom present. Scores ≥ 3 were defined as symptom present, and scores ≥ 5 as symptom present and severe. These cut points have been used in similar studies [8-11] and are also in accordance with official Norwegian recommendations [4].

Ward staff recorded the following background information on the forms: age group, gender, main diagnoses (maximum three), and number of days in hospital.

Patients were excluded from the study if they were cognitively impaired or otherwise unfit to participate, if they had had surgery the preceding day, or if they were unwilling to participate.

Patients were defined as "palliative" if they had an advanced, serious, chronic disease with limited life expectancy and symptom relief as the main goal of treatment. This categorization was denoted by attending staff on the wards, nurses and/or physicians. In case of doubt, the staff was told to ask themselves the following question, "Would you be surprised if this patient died in the course of the next nine months?" If the answer was no, the patient could be defined as palliative.

The study was approved by the Regional Committee for Medical Research Ethics and the Privacy Issues Unit of the Norwegian Social Science Data Services.

## Results

### All patients

**Figure 1:**
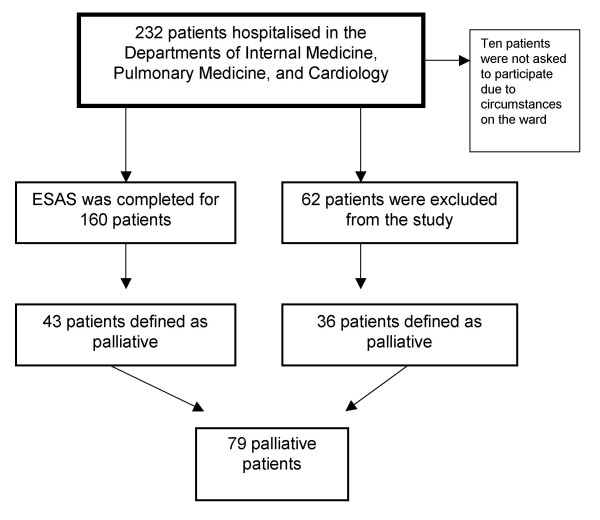
Participant distribution in the study.

Altogether 232 patients were recorded as inpatients on the wards in question on the day of the study. A total of 222 patients were registered in the study (Fig. 1). Ten patients were not asked to participate due to circumstances on their ward and lack of time for the staff.

Sixty-two patients were excluded from the study due to the following reasons: 32 patients were not able to participate, 19 patients were not willing to participate, six patients had undergone surgery in the last 24 hours, four patients were not able to complete the questionnaire or the interview (drop outs), and one patient was in a state in which it was deemed unethical to ask for participation.

ESAS was completed for 160 patients. In 105 cases (66%) the ESAS was completed by the patients themselves. In the remaining 55 cases (34%) the questionnaire was filled in by the staff by interview.

More than 50% of the included patients reported fatigue (61%), dry mouth (53%), and lack of wellbeing (56%), and 49% reported dyspnea and lack of appetite (NRS ≥ 3). Within the group reporting symptoms, more than half of the patients rated their symptoms as severe (NRS ≥ 5). Pain at rest was reported by 34% of all included patients, and pain on movement by 43%. Eighteen % of the patients reported nausea.

With respect to diagnosis, the study population was very heterogeneous, with 54% having more than one main diagnosis. The diagnostic group heart and vascular diseases (N = 52) covered a variety of diagnoses with different symptom profiles. Chronic obstructive lung disease (COPD) patients (N = 27) were a much more uniform group, presenting high scores for dyspnea and lack of wellbeing. In the COPD group, 81% of the patients rated their dyspnea as severe (NRS ≥ 5), and 46% reported a severe lack of well-being, compared to 25% in the group not having this diagnosis.

**Figure 2:**
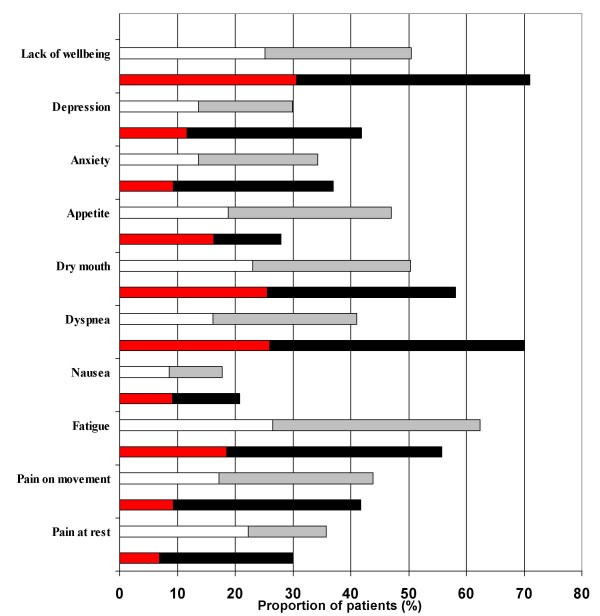
**Comparison of symptoms between the palliative patient subgroup (N = 43) and the non-palliative group (N = 117)**. The red and black columns show the palliative patient subgroup; the red columns show the proportion of palliative patients with NRS score 3–4 for each symptom, and the black columns show the proportion of palliative patients with NRS score ≥ 5 for each symptom. The white columns show the proportion of non-palliative patients with NRS 3–4 for each symptom, and the grey columns show the proportion of non-palliative patients with NRS ≥ 5 for each symptom.

Among the 160 included patients, 117 were denoted as non-palliative. Their characteristics are shown in Table 1, and the symptom distribution in this group is presented in Fig. 2. Fatigue was the most prevalent symptom. There were 13 cancer patients in the non-palliative group. The cancer patients had more pain and nausea than the patients with other diagnoses, while dyspnea was more common in the non-cancer patients.

**Table 1:** Characteristics of the study population, comparing the palliative and the non-palliative group.

	**Palliative group**		**Non-palliative group**		
	**Included ** N = 43 (19)^#^	**Excluded ** N = 36 (16)	**Included ** N = 117 (53)	**Excluded ** N = 26 (12)	**All patients ** N = 222*

**Gender**					
Female	16 (37.2)	16 (44.4)	61 (52.1)	11 (42.3)	104 (46.8)
Male	27 (62.7)	20 (55.6)	56 (47.9)	15 (57.7)	118 (53.1)
**Age groups**					
Age < 31	-	-	13 (11.1)	3 (11.5)	16 (7.2)
Age 31–40	1 (2.3)	-	12 (10.3)	4 (15.4)	17 (7.7)
Age 41–50	2 (4.7)	-	17 (14.5)	2 (7.7)	21 (9.5)
Age 51–60	1 (2.3)	-	23 (19.7)	4 (15.4)	28 (12.6)
Age 61–70	9 (20.9)	2 (5.6)	20 (17.1)	6 (23.1)	37 (16.7)
Age 71–80	19 (44.2)	10 (27.8)	23 (19.7)	4 (17.4)	56 (25.2)
Age 81–90	9 (20.9)	18 (50.0)	7 (6.0)	3 (11.5)	37 (16.7)
Age > 91	2 (4.7)	6 (16.7)	2 (1.7)	-	10 (4.5)
**Number of diagnoses**					
One diagnosis	12 (27.9)	11 (30.6)	62 (53.0)	15 (57.7)	100 (45.0)
Two diagnoses	15 (34.9)	12 (33.3)	36 (30.8)	7 (27.0)	70 (31.5)
Three diagnoses	16 (37.2)	13 (36.1)	19 (16.2)	4 (15.4)	52 (23.4)
**Diagnosis**					
Renal failure	3 (6.9)	1 (2.8)	6 (5.2)	3 (11.5)	13 (5.9)
COPD	14 (32.6)	5 (13.9)	13 (11.1)	1 (3.8)	33 (14.9)
Heart and vascular diseases	21 (48.9)	15 (41.7)	31 (26.5)	6 (23.1)	73 (32.9)
Cancer	16 (37.2)	3 (8.3)	13 (11.1)	2 (7.7)	34 (15.3)
**Mean days in hospital**	10.6	11.5	8.8	11.8	9.9

^# ^Percentages are given in brackets. Percentages given in the column headings and the column to the far right relate to the total number of patients (222), while percentages given for each of the four subgroups relate to the number of patients within that group.

*232 patients were recorded as inpatients on the day of the study. Ten patients were not asked to participate due to circumstances on their ward.

### The palliative patient subgroup

Seventy-nine patients (35.6%) were defined as palliative by the attending staff on the wards (Fig. 1). Their diagnoses were denoted by the attending staff and are presented in Table 1.

Forty-three of the palliative patients were included in the study, and 36 were excluded.

Altogether, there were more male than female patients in the palliative group, and almost twice as many males from the palliative group were included in the study. The included and excluded palliative patients had very similar mean and median lengths of stay.

The patients in the palliative group were older than the ones not denoted as palliative, with 80% of the palliative patients above 70 years of age, and 40% above 80.

The palliative patients that were excluded from the study mainly belonged to the highest age group (>80 years).

The average total ESAS score (symptom distress score) for the included palliative patients was 29.5 (range 6–83).

The symptom distribution within the palliative group is shown in Fig. 2. Seventy percent of the palliative patients reported dyspnea and lack of wellbeing. Other symptoms reported by this group were dry mouth (58%), fatigue (56%), depression (41%), anxiety (37%), pain at rest (30%), and pain on movement (42%). Fig. 2 shows that the majority of palliative patients reporting symptoms rated their symptoms as severe. Almost 45% of the included palliative patients reported severe dyspnea, and 40% a severe lack of wellbeing. Twenty-three % of the palliative patients reported severe pain at rest and one third of the group severe pain on movement. Thirty percent of the included patients in the palliative group felt severely depressed. The palliative patients reported more severe dyspnea, more depression, and a greater lack of wellbeing than the non-palliative patients did (Fig. 2). Within the palliative subgroup, the 16 cancer patients reported a greater lack of wellbeing than non-cancer patients, while patients with non-cancer diagnoses reported more severe dyspnea.

## Discussion

This cross-sectional study shows that more than one third of the patients in a Department of Internal Medicine were defined as palliative, and that the majority of the patients in this palliative group reported distressing symptoms.

In many cases it proved difficult for the staff to decide whether the patient should be regarded as "palliative" or not. Generally, the nurses and doctors were restrictive in their use of the term "palliative". Several patients were added to the palliative group when we asked our question "Would you be surprised if this patient died in the course of the next nine months?" When looking through the completed forms afterwards, we noted several patients whom we clearly would have defined as palliative, but who were not marked as such. However, in order not to introduce any bias, we did not alter anything. We therefore think that the number of palliative patients reported here is underestimated.

A large proportion of patients in the palliative group were excluded from the study due to cognitive impairment or because their general condition made them unfit to participate. This led to a reduced inclusion rate in the palliative subgroup. However, the high age and poor general condition of these patients add additional evidence that this group constituted a palliative care population.

In a recent review of symptom prevalence in advanced, life-threatening disease, the three symptoms pain, breathlessness, and fatigue were described among more than 50% of patients with advanced cancer, AIDS, heart disease, COPD, and renal disease [12]. This is consistent with our findings for dyspnea (70%) and fatigue (56%), which were the dominating symptoms in the present study. Solano and coworkers reported a pain prevalence of between 34% and 77% in heart disease and COPD in different studies [12]. Pain on movement was reported by 43% in our palliative group. The prevalence of depression in our study is also consistent with other reports [12].

According to Norwegian recommendations we used the value 3 on the numerical rating scales as cut-off point for symptom presence [4]. This is more restrictive than in several other studies and may influence the prevalence numbers [6,9,11].

This study demonstrates that patients in a Department of Internal Medicine have a high prevalence of distressing symptoms and that there consequently is a need for skills in symptom control on the ward. A thorough assessment is a prerequisite for successful symptom relief. We used a modified ESAS questionnaire in our study [4,5]. The ESAS is an example of a quick and clinically relevant symptom assessment tool. Although developed for palliative care cancer patients, the tool covers a range of symptoms which also commonly is present in advanced non-malignant disease [12]. Routine use of ESAS is a help to reveal new symptoms, prioritize between symptoms, and repeatedly evaluate the degree of symptom control obtained. However, symptom assessment is particularly challenging in some subgroups. These include cognitively impaired patients, patients whose language or culture differs from that of the health care professionals, and the imminently dying. The present study confirms that many patients in a palliative care population are too weak to use self-rating assessment tools, and that other approaches must be used. Family members and staff may be useful proxies for clinical and research purposes, but their data must be interpreted cautiously [11,13,14]. Numerous studies have demonstrated that observer and patient assessments are not highly correlated [11,13-15]. For non-verbal patients with severe cognitive impairment, pain assessment must rely on behavioural scales [16]. Although some observational tools exist, additional research is needed regarding instruments for symptom assessment in patients who cannot give subjective ratings.

Although the situation varies between the countries, the majority of palliative care services in Europe have cancer patients as their main target population [17]. In Norway, more than 90% of the patients in specialist palliative care are cancer patients [4]. However, our study reveals considerable palliative care needs in an inpatient population of a general medical department, representing an unselected, heterogeneous patient population. These findings clearly have implications for the planning and development of the palliative care services in our hospital. First of all, the attending staff on the wards must possess skills in palliative care to make a valid symptom assessment and provide basic symptom relief. In addition, there is a need for specialist palliative care providers with experience in non-cancer care to work in close cooperation with the ward staff. We also think that the present findings are representative of Internal Medicine Departments in many hospitals, both in Norway and abroad, and may even apply to Surgical services and Neurology Departments. In many hospitals, all of these departments mainly care for patients in the older age group, with advanced disease and significant comorbidities.

The present study only focused on symptom prevalence as measured by the ESAS, which is an obvious limitation with respect to needs assessment. Understanding needs outside of symptoms, like spiritual challenges, family and carer needs, and coordination of services, forms a natural part of palliative care and should be included in a complete survey. However, even if the evidence is limited, previous studies have shown that e.g. family anxiety and lack of psychological support are prevalent also in non-cancer [18]. Given the symptom burden, we do not anticipate that spiritual, social, or psychological needs are very different in a non-cancer population compared to a cancer population in the last year of life.

Comprehensive palliative care successfully improves the quality of life for cancer patients and families, and has also been shown to reduce the overall cost of care by reducing the amount of time spent in acute hospital settings [19]. This option must be expanded to patients with other advanced, progressive, life-threatening diseases. Providing palliative care for advanced, chronic non-malignant disease may be more challenging than for cancer patients due to difficulties related to establishing a prognosis or identifying the patients' needs [18,20]. However, these challenges should not prevent suffering patients from getting optimal symptom relief. Palliative care should not only be offered by specialist teams or units when all other treatments have failed, but be an integral part of good patient care in any setting.

## Conclusion

More than one third of the patients in a Department of Internal Medicine were defined as palliative, and the majority of the patients in this palliative group reported severe symptoms. There is a need for skills in symptom control on medical wards. Services should be available on the basis of need in terms of symptoms and problems rather than on the basis of diagnosis.

## Competing interests

The authors declare that they have no competing interests.

## Authors' contributions

Both authors made substantial contributions to the intellectual content of the manuscript and have approved the final version. KRS initiated the study and had primary responsibility for analysing the data and writing the paper. DFH participated in the planning of the study and the data collection and contributed critical insights in interpretation of the data. KRS is the guarantor.

## Pre-publication history

The pre-publication history for this paper can be accessed here:


